# Art of imaging: Aurora Cerebralis Rivers of the Mind

**DOI:** 10.1093/radadv/umag012

**Published:** 2026-03-04


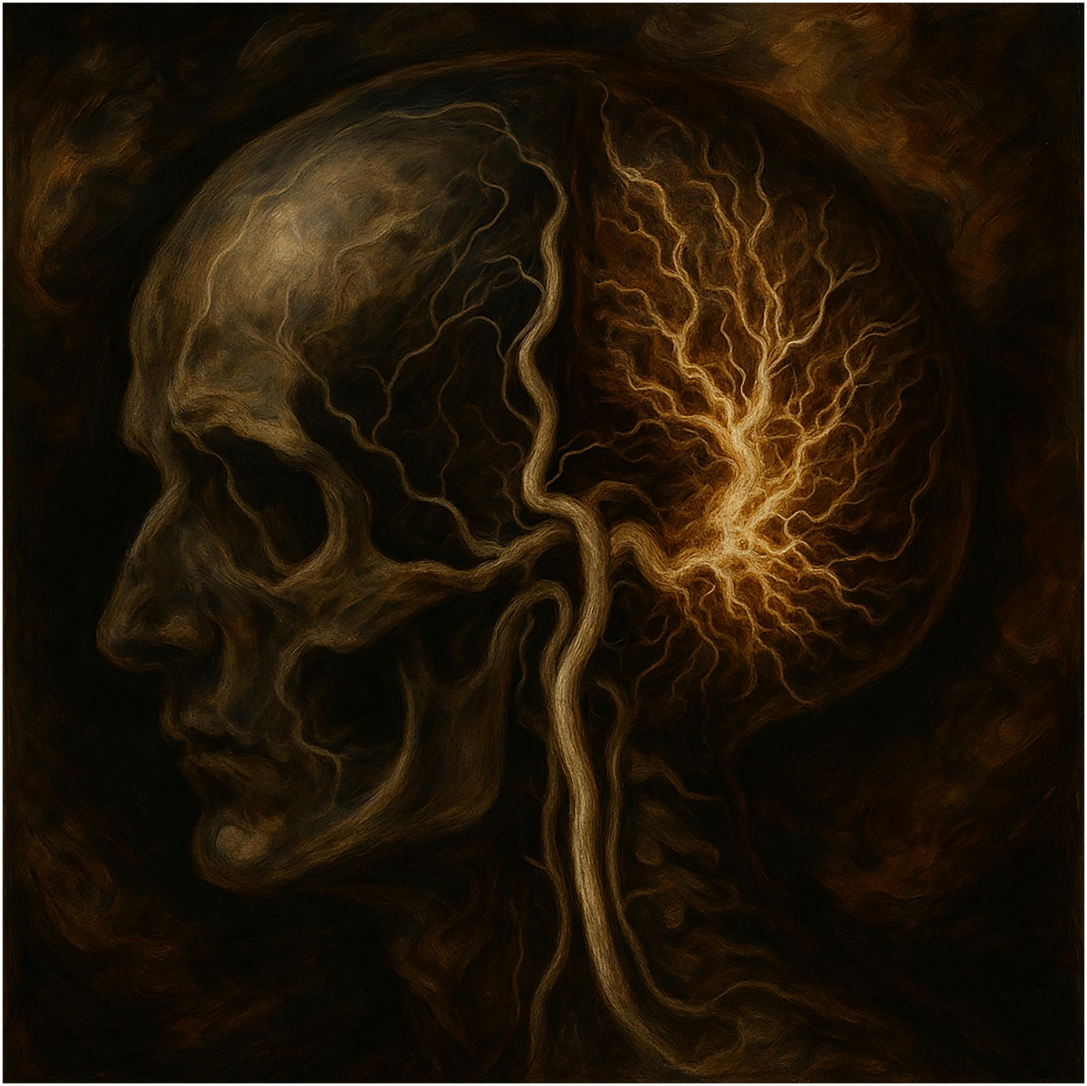


The brain emerges as both battleground and masterpiece—a place where beauty and struggle become inseparable


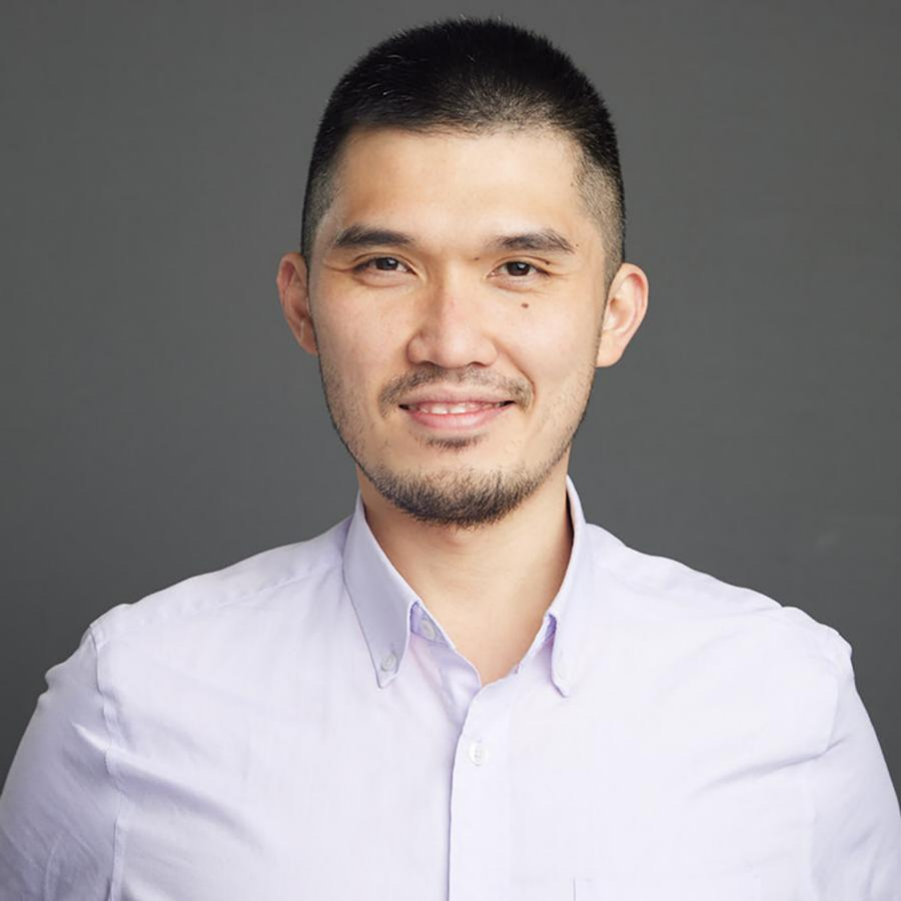


Fatt Yang Chew, MD

Neuroradiologist & Interventional Neuroradiologist, China Medical University Hospital, Taichung, Taiwan, driven by innovation and purpose.

